# Community profiling of the intestinal microbial community of juvenile Hammerhead Sharks (*Sphyrna lewini*) from the Rewa Delta, Fiji

**DOI:** 10.1038/s41598-019-43522-x

**Published:** 2019-05-09

**Authors:** Natacha M. S. Juste-Poinapen, Lu Yang, Marta Ferreira, Johann Poinapen, Ciro Rico

**Affiliations:** 1 The University of the South Pacific, Molecular Analytics Laboratory (MOANA LAB), School of Marine Sciences, Suva, Fiji; 2 JNP Ltd, Suva, Fiji; 3 The University of the South Pacific, The Institute of Applied Sciences, Suva, Fiji; 4 Singapore Centre for Environmental Life Sciences Engineering, Nanyang Technological University, Singapore, 637551 Singapore; 5 CIIMAR/CIMAR—Interdisciplinary Centre of Marine and Environmental Research, University of Porto, Terminal de Cruzeiros do Porto de Leixões, Av. General Norton de Matos s/n, 4450-208 Matosinhos, Portugal; 6 Instituto de Ciencias Marinas de Andalucía (ICMAN), Consejo Superior de Investigaciones Científicas, Campus Universitario Río San Pedro, 11510 Puerto Real, Cádiz, Spain

**Keywords:** Microbiology, Microbial ecology

## Abstract

Fourteen juvenile scalloped hammerhead sharks (*Sphyrna lewini*; SHS) were captured between November and December 2014 in the Rewa Delta in Fiji, and assessed for intestinal microflora characterisation using 16S rRNA amplicon sequencing by Illumina Miseq. The microbial population revealed a fluctuating dominance between the Enterobacteriaceae and *Vibrionaceae* families, namely *Citrobacter* and *Photobacterium* spp. Other related marine operational taxonomic units were closely related to *Afipia felis*, *Chloroflexus aggregans*, *Psychrobacter oceani*, *Pontibacter actiniarum* and *Shigella sonnei*. Two sharks had distinctive profiles that were dominated by known pathogens, namely *Aeromonas salmonicida* and *Klebsiella pneumonia*. The presence of a *Methanosaeta* species, and of *Shigella* and *Psychrobacter*, would suggest sewage contamination because of a spill that occurred on the 6^th^ of December 2014. This study successfully establishes a baseline for future research.

## Introduction

The scalloped hammerhead shark (SHS), *Sphyrna lewini*, is a circumglobally distributed large apex predator species common to tropical and warm temperate coastal and semi-pelagic marine environments. Due to overfishing and habitat destruction, the species is considered to be among the most globally threatened sharks and was declared endangered by the International Union for Conservation of Nature (IUCN) Red List in 2007^1^. It is also currently listed on Appendix II of the Convention on International Trade in Endangered Species of Wild Fauna and Flora (CITES)^2^. Several SHS populations have been heavily exploited worldwide by both inshore and offshore fisheries^3^.

**Figure 1:**
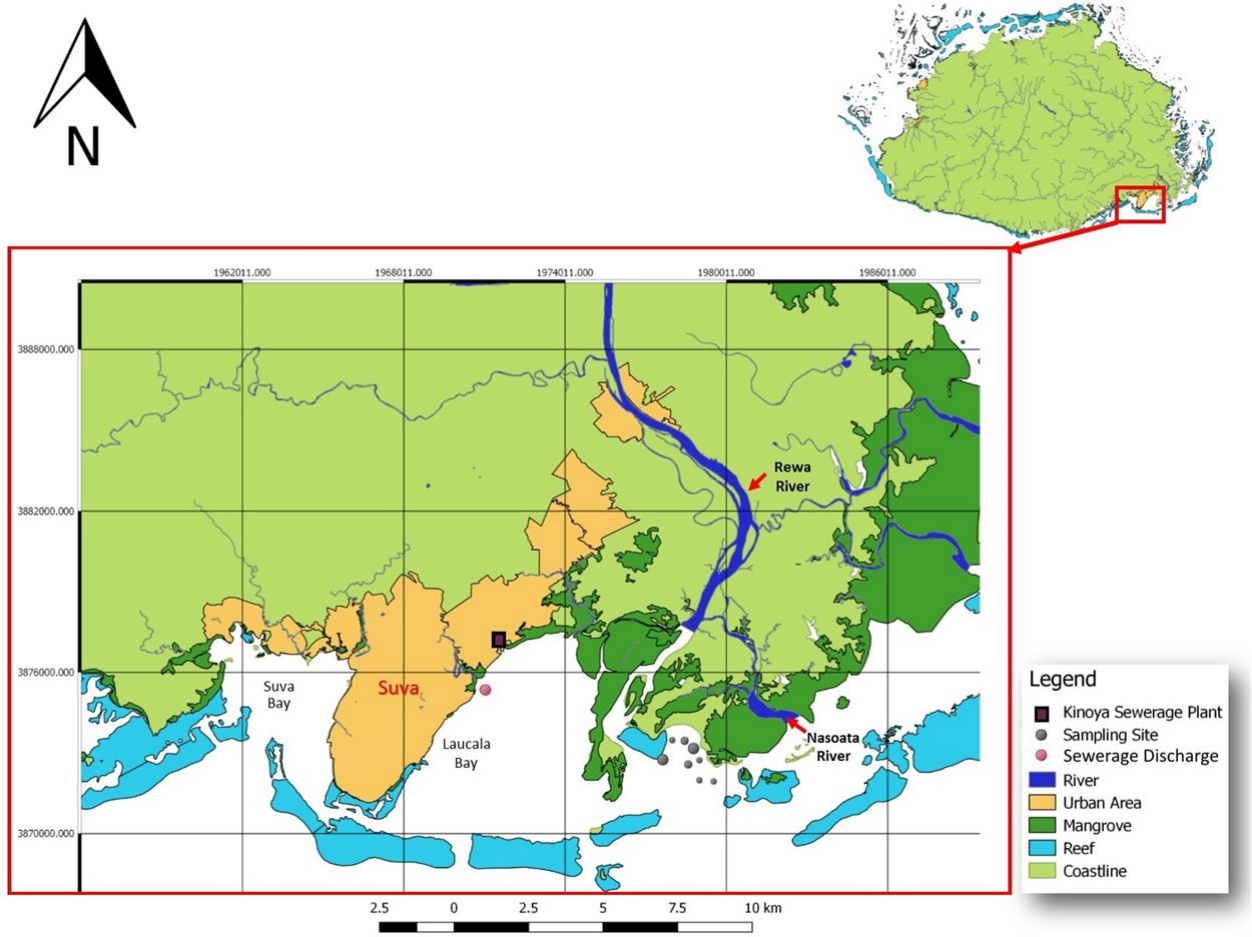
Geographical location of sampling sites in the Rewa Delta, and location of sewage discharge during the spillage that occurred in December 2014, Viti Levu, Fiji (The map has been prepared by Mr Sione Kaituu using online resources from QGIS, version 3.6.0 https://www.qgis.org/en/site/). Grey dots reflect specific places where samples were collected, and the diameter is proportional to the number of sharks taken at that particular site.

In Fiji waters, the species has been reported to aggregate in the Austral summer in seven estuarine areas on the main islands of Viti and Vanua Levu^4^. Marie, *et al*.^5^, recently confirmed that the Rewa Delta is a critical habitat for SHS by documenting its year-round presence. However, they found significant seasonality of occurrence and abundance in the area, with a parturition period during the wet austral spring and summer seasons between October and March with a population peak between December and February. Subsequently, Vierus, *et al*.^6^ showed that neonates and juveniles of SHS also aggregate in the Ba estuary during the same period. The evidence presented in these studies unequivocally suggest the need for developing and strengthening shark conservation and management measures within Fiji, as several estuaries may well be nursery areas for SHS. As is common for estuaries, the Rewa Delta (Fig. 1) is characterised by large fluctuating salinities, a fresh water layer, especially during the Austral summer, high turbidity, and an abundance of small crustaceans, fish, eels, and shellfish^7^. These conditions are characteristic of shark nurseries, where high levels of primary production increase population productivity and enhance young sharks’ chances of survival^5,8,9^.

Detailed knowledge of SHS ecology, behaviour and habitat requirements, particularly during the first stage of their life, is still limited^10^. In addition, there are indications that contamination by human and animal waste from villages lining the riverbanks may have shifted the balance of the ecosystem of the Suva Lagoon by increasing nutrient and bacterial load in water and organisms^11–13^. The main contributors of nutrients in Laucala Bay associated with sewage are the effluent of the Kinoya sewage treatment plant released at the sea outfall located in Laucala Bay (Fig. 1), and the human and animal waste from villages on the banks of the Rewa River^12^. Fish and most shellfish are directly affected by degraded environments through their static feeding behaviour, while juvenile scallop hammerhead sharks experience low abundance of food or consume contaminated prey^14^.

Fish diseases caused by enteric bacteria have been reported in eutrophic waters associated with faecal pollution^15^, thus sharks feeding on prey living in sewage-polluted waters would reflect the bacterial load present in those waters. Studying the effect of the microbiome in conjunction to other factors is important in evaluating the environmental quality of critical habitats of endangered species. There are many ways to monitor pollution in the marine ecosystem, one of which is the use of indicator microorganisms. For example, faecal coliform such as *Escherichia coli* are indicators of contamination of water with faecal matter from humans or warm-blooded animals. This further implies that other pathogenic bacteria belonging to species of *Salmonella*, *Shigella*, *Pseudomonas*, and *Streptococcus* can also be present^14,16^. The intestine constitutes an ideal niche for microorganisms due to its readily available source of carbon, minerals, and solutes that are conducive to growth. Because, there is evidence that the microbial colonisation of the intestine of vertebrates starts after hatching or birth, it is influenced by the environmental factors that surround the habitat where a newly hatched or born individual begins to feed^17^. Consequently, investigation of bioindicators in aquatic animals usually involves the characterisation of the intestinal microbial profile at different life stages^17,18^.

For this bioindicator method to be successful, a baseline needs to be established to differentiate between normal colonisers and potential pathogens. Emerging molecular methods for analysing microbial communities allow high-resolution assessments of complex communities. Such protocols usually include culture-independent microbial profiling based on 16S ribosomal RNA (16S rRNA), which is not limited by cultivability, and can often detect even the least abundant members of the microbial community^19^. To the best of our knowledge, no data on the intestinal microbial profile of scallop hammerhead sharks are available in the literature, while studies on the gut microbiome of other shark species are limited. Exceptions are for striped burrfish spinner sharks (*Carcharhinus brevipinna*), atlantic sharp nose sharks (*Rhizoprionodon terraenovae*), and sandbar sharks (*Carcharhinus plumbeus*)^20–23^.

As part of ongoing projects to understand and protect critical shark habitats in Fiji, this study generates baseline data about the intestinal microbial profile of a representative sample of SHS from the Rewa Delta using 16S rRNA Illumina MiSeq amplicon sequencing (MiSeq). The database thus generated should contribute to the reference library of intestinal colonisers of juvenile SHS and possibly support further studies on trends in microbiological communities and the identification of bioindicator microorganisms as impacted by pollution of the waterways.

## Results

### Bacterial diversity profile

**Figure 2:**
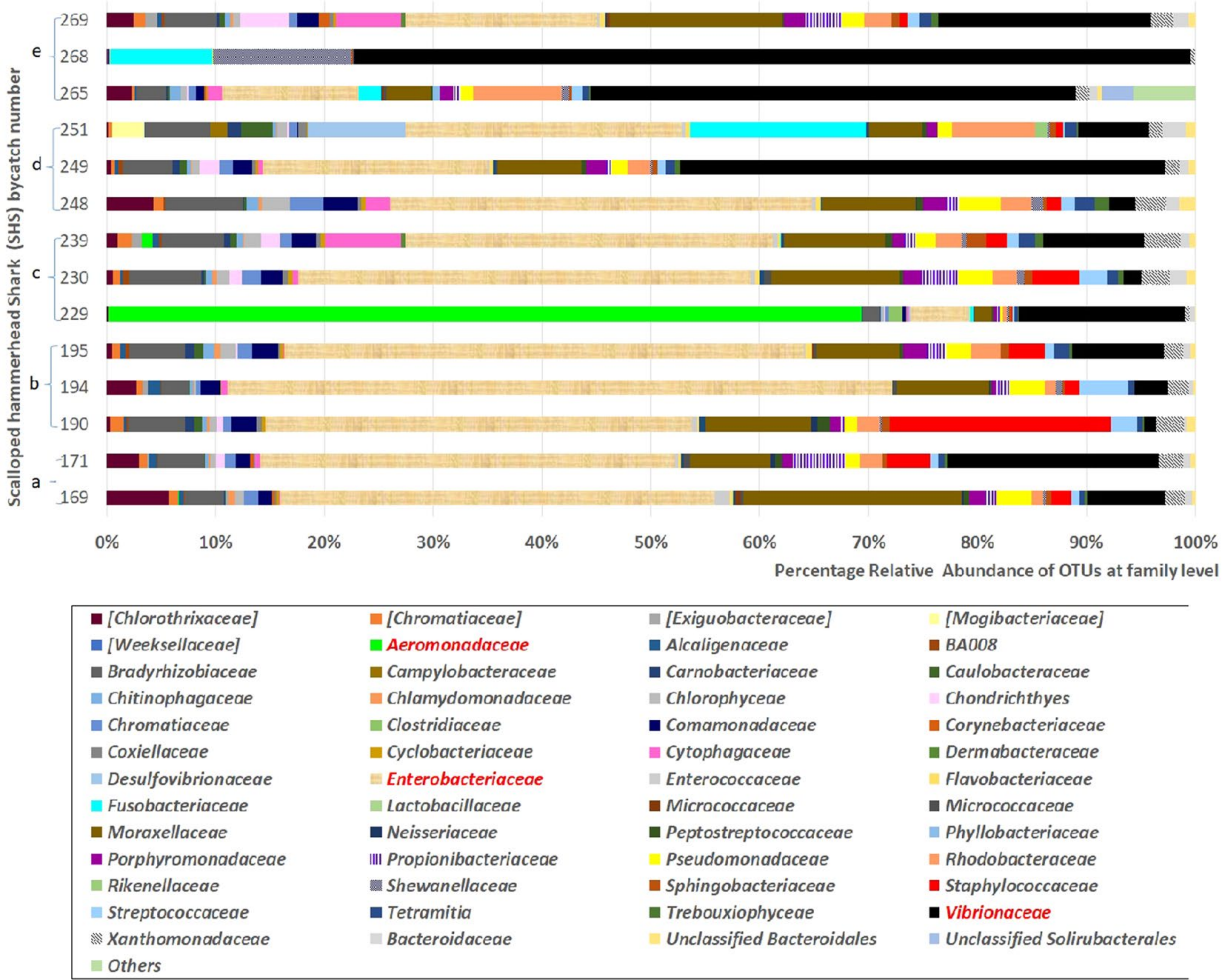
Bacterial community profiles, including percentage relative abundances of the all identified taxonomic bacterial groups specified to the family level, found in selected juvenile hammerhead sharks captured in 2014 on the (**a**) 20–21 November, (**b**) 1 December, (**c**) 8–9 December. (**d**) 12 December and (**e**) 22 December, in the Rewa delta. The 2 OTUs with the highest relative abundance (*Enterobacteriaceae* and *Vibrionaceae*) and the OTU highly distinguished in SHS 229 (*Aeromonadaceae*) are highlighted in red in the figure legend.

Since ANOSIM indicated that the variability among the technical repeats was negligible (R = 0.602, P = 0.001), an average was used to compute the bacterial diversity of each shark, which will be referred to by their catch number. The major microbial communities were identified to the family level and analysed for percentage relative abundance, with greater focus given to those that make at least 10% of the community in a minimum of one shark (Fig. 2**)**. *Enterobacteraceae* was detected in high abundance in most sharks, while *Vibrionaceae* was seen to be more dominant in most sharks collected after the 9^th^ of December 2014, namely SHS: 251 (44.2%), 265 (43.6%), 268 (17.5%) and 269 (76.0%). Samples from another shark had a distinctive profile: SHS 229 demonstrated an average of 68.8% relative abundance of *Aeromonadaceae*. The Shannon Index (1.07 ± 0.51) and Simpson index (0.39 ± 0.02) for SHS 229 was also lower in comparison with the other samples (Table 1), with a Chao 1 estimates of 4.67 ± 0.67, indicating less species richness and evenness. Similar observations were made for SHS 268 (Shannon Index:1.12 ± 0.61, Simpson Index: 0.37 ± 0.04 and Chao 1 estimate: 5.50 ± 0.50) and SHS 249 (Shannon Index:1.73 ± 0.73, Simpson Index: 0.50 ± 0.11 and Chao 1 estimate: 10.33 ± 2.18). A more diverse and evenly distributed microbial profile was attributed to SHS 251 (Shannon Index: 3.29 ± 0.10, Simpson Index: 0.85 ± 0.04 and Chao 1 estimate: 23.25 ± 5.41). In addition, members of the *Moraxellaceae, Bradyrhizobiaceae, Pseudomonadaceae, Rhodobacteraceae, Staphylococcaceae*, and *Streptococcaceae* were also detected among these samples.

**Table 1:** Comparison of the alpha diversity indices, based on natural log (QIIME, Version 1.8.0), including Shannon and Simpson indices as well as Chao 1 estimates, for each of the SHS studied, based on the average of triplicate samples per specimen over a time series.

Hammerhead shark reference (SHS)	Date captured	Shannon Index (±standard error)	Simpson Index (±standard error)	Chao 1 (±standard error)
169	20 Nov 2014/0	2.40 ± 0.01	0.68 ± 0.04	24.33 ± 8.33
171	21 Nov 2014/1	2.57 ± 0.11	0.74 ± 0.02	14.08 ± 1.24
190	1 Dec 2014/11	3.11 ± 0.40	0.80 ± 0.05	26.00 ± 3.47
194	1 Dec 2014/11	2.33 ± 0.23	0.64 ± 0.08	21.50 ± 4.82
195	1 Dec 2014/11	2.40 ± 0.43	0.71 ± 0.05	19.70 ± 3.18
229	8 Dec 2014/18	1.07 ± 0.51	0.39 ± 0.02	4.67 ± 0.67
230	8 Dec 2014/18	2.78 ± 0.14	0.72 ± 0.03	29.83 ± 7.66
239	9 Dec 2014/19	2.65 ± 0.25	0.76 ± 0.02	24.28 ± 4.02
248	12 Dec 2014/22	2.68 ± 0.52	0.72 ± 0.15	26.75 ± 8.71
249	12 Dec 2014/22	1.73 ± 0.73	0.50 ± 0.11	10.33 ± 2.18
251	12 Dec 2014/22	3.29 ± 0.10	0.85 ± 0.04	23.25 ± 5.41
265	22 Dec 2014/32	2.44 ± 0.29	0.68 ± 0.05	26.17 ± 5.93
268	22 Dec 2014/32	1.12 ± 0.61	0.37 ± 0.04	5.50 ± 0.50
269	22 Dec 2014/32	3.09 ± 0.03	0.84 ± 0.01	13.00 ± 0.50

A higher value indicates a greater diversity.

**Figure 3:**
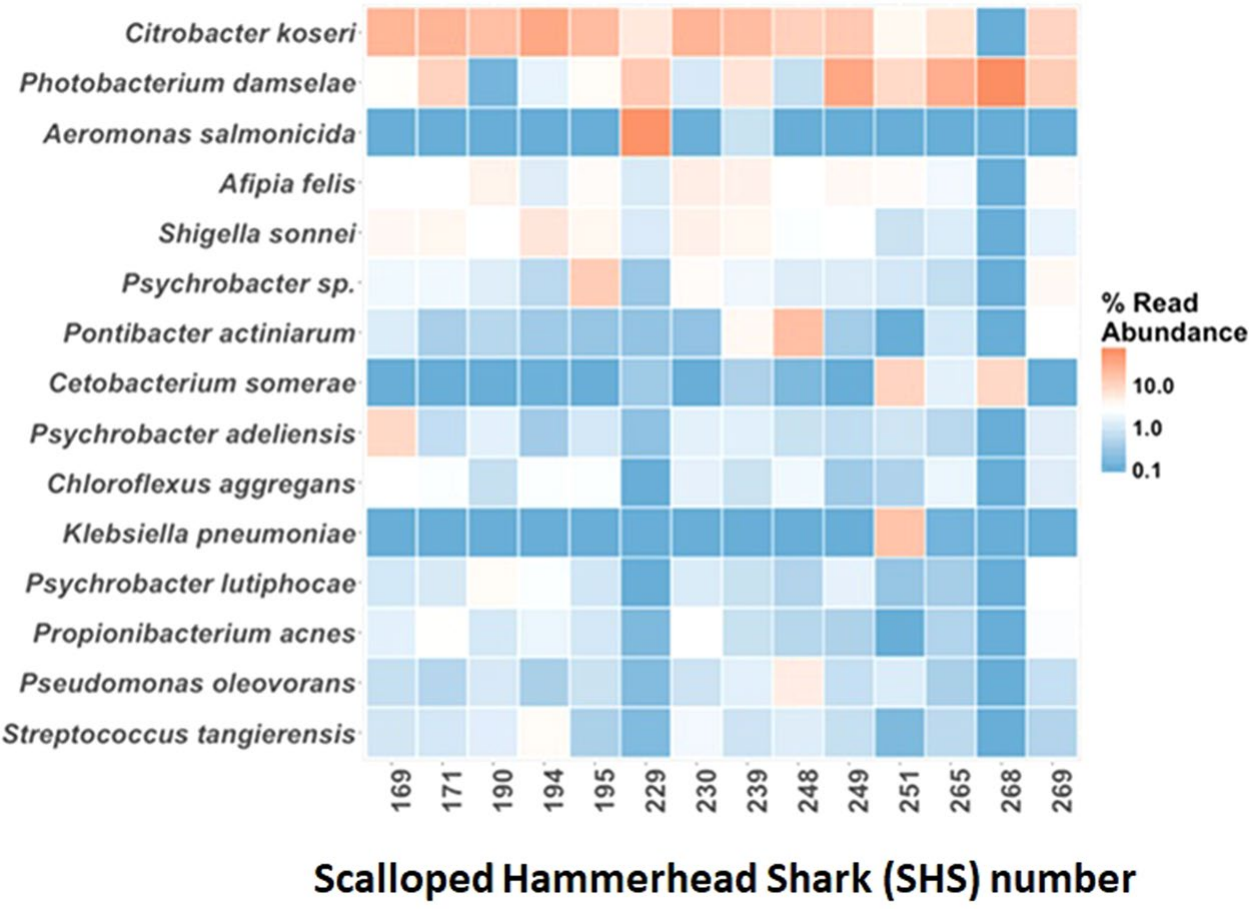
Heatmap of intestinal microbial communities in sharks with top 15 OTUs (relative abundance of at least 10% in a minimum of one sample). Samples are identified by their SHS capture number.

Further comparison between the microbial profiles of the juvenile SHS sharks is illustrated in a Heatmap (Fig. 3). The *Enterobacteriaceae* microbes were further classified with major species of the genera *Citrobacter*, *Shigella and Klebsiella*. A bacterium closely related to the *Citrobacter koseri* (Accession No: HQ992945.1) was the most prevalent in most samples, ranging from a highest percentage relative abundance of 41.0% in SHS 194 to as low as 0.11% in SHS 268. While the presence of *Shigella* sp. (Accession No: KF225561.1) was observed in all samples at varying percentages, the other member of the *Enterobacteriaceae* family, associated with *Klebsiella pneumoniae* (Accession No: CP003200.1), was detected at highest relative abundance (19.9%) only in SHS 251. The data further suggested that the *Vibrionaceae* family is comprised mostly of *Photobacterium* spp. Other species associated with the top 15 operational taxonomic units (OTUs) identified in this experiment were *Afipia* spp. (*Bradyrhizobiaceae*), *Cetobacterium* spp. (*Fusobacteriaceae*), *Chloroflexus* spp. (*Chlorothrixaceae*), *Psychrobacter* spp. (*Moraxellaceae*) and *Propionibacterium* spp. (*Propionibacteriaceae*).

Compilation of the closely related species to the top five OTUs, in order of highest percentage relative abundance for each SHS (Table 2), indicated that either a *Citrobacter* sp. or a *Photobacterium* sp. was the top OTU. Also, commonly detected were *Shigella* spp. *Afipia* spp. and *Chloroflexus* spp. This analysis also revealed distinctive populations in three SHS, including SHS 229, where the top OTU was *Aeromonas* spp. (68.8%). As already mentioned, *Klebsiella* sp. (19.9%) was seen in SHS 251, which also had *Cetobacterium* sp. (16.6%) as the second most relative abundant species. The emergence of a *Shewanella* sp. (10.5%) was observed in SHS 268, the microbial profile of which was dominated by *Photobacterium* sp. (76.0%). *Staphylococcus* and *Streptococcus* species were also detected, but in lower relative abundance. For example, among the top 10 OTUs, a *Staphylococcus* sp. closely related to *Staphylococcus warneri* (Accession No: KY623039.1) was linked to SHS: 169 (1.0%), 171 (1.0%) and 194 (1.6%). Similarly, a *Streptococcus* sp. linked to *Streptococcus tangierensis* (Accession No: KF999656.1) was shown in profiles for SHS 190 (4.4%), 194 (2.4%), and 230 (2.5%).

**Table 2:** Top 5 OTUs closely related species (100% sequence identity), identified with BLASTn of 16S rRNA sequences generated by Illumina Miseq.

SHS number	Time of bycatch	Top 5 OTUs closely related species (% relative abundance)
169	20 Nov 2014	^1^*Citrobacter koseri* (33.3), ^2^*Psychrobacter adeliensis* (12.9), ^3^*Photobacterium damselae* (6.5), ^4^*Chloroflexus aggregans* (5.6), ^5^*Shigella sonnei* (5.4)
171	21 Nov 2014	^1^*Citrobacter koseri* (32.3), ^3^*Photobacterium damselae* (13.8), ^6^*Vibrio proteolyticus* (5.3), ^5^*Shigella sonnei* (5.1), ^7^*Propionibacterium acnes* (4.8)
190	1 Dec 2014	^1^*Citrobacter koseri* (50.7), ^5^*Shigella sonnei* (7.5), ^8^*Streptococcus tangierensis* (4.4), ^9^*Enhydrobacter aerosaccus* (2.9), ^10^*Brevundimonas nasdae* (2.8)
194	1 Dec 2014	^1^*Citrobacter koseri* (32.3), ^11^*Macrococcus caseolyticus* (12.6), ^12^*Psychrobacter oceani* (6.5), ^13^*Afipia felis* (5.3), ^5^*Shigella sonnei* (4.3)
195	1 Dec 2014	^1^*Citrobacter* koseri (41.0), ^3^*Photobacterium* damselae (8.0), ^5^*Shigella sonnei* (6.0), ^13^*Afipia felis* (5.2), ^9^*Enhydrobacter aerosaccus* (1.9)
229	8 Dec 2014	^14^*Aeromonas salmonicida* (68.8), ^3^*Photobacterium damselae* (15.0), ^1^*Citrobacter koseri* (4.3), ^13^*Afipia felis* (1.5), ^5^*Shigella sonnei* (0.8)
230	8 Dec 2014	^1^*Citrobacter koseri* (33.6), ^13^*Afipia felis* (6.6), ^5^*Shigella sonnei* (6.2), ^12^*Psychrobacter oceani* (4.3), ^7^*Propionibacterium acnes* (3.4)
239	9 Dec 2014	^1^*Citrobacter koseri* (27.4), ^3^*Photobacterium damselae* (8.1), ^15^*Pontibacter actiniarum* (7.0), ^13^*Afipia felis* (5.6), ^5^*Shigella sonnei* (5.1)
248	12 Dec 2014	^1^*Citrobacter koseri* (32.1), ^13^*Afipia felis* (6.9), ^5^*Shigella sonnei* (5.4), ^4^*Chloroflexus aggregans* (4.2), ^16^*Pectinodesmus pectinatus* (2.5))
249	12 Dec 2014	^3^*Photobacterium damselae* (44.3), ^1^*Citrobacter koseri* (17.2), ^13^*Afipia felis* (4.6), ^5^*Shigella sonnei* (3.1), ^12^*Psychrobacter oceani* (2.3)
251	12 Dec 2014	^17^*Klebsiella pneumoniae* (19.9), ^18^*Cetobacterium somerae* (16.6), ^13^*Afipia felis* (6.1), ^19^*Desulfonatronum lacustre* (6.1), ^3^*Photobacterium damselae* (5.6)
265	22 Dec 2014	^3^*Photobacterium damselae* (43.6), ^1^*Citrobacter koseri* (10.3), ^20^*Gemmobacter nectariphilus* (3.5), ^21^*Sulfitobacter mediterraneus* (3.4), ^22^*Solirubrobacterales bacterium* (2.9)
268	22 Dec 2014	^3^*Photobacterium damselae* (76.0), ^23^*Shewanella baltica* (10.5), ^18^*Cetobacterium somerae* (9.3), ^24^*Shewanella piezotolerans* (2.0), ^6^*Vibrio proteolyticus* (0.5)
269	22 Dec 2014	^3^*Photobacterium damselae* (17.5), ^1^*Citrobacter koseri* (14.3), ^15^*Pontibacter actiniarum* (6.1), ^13^*Afipia felis* (4.8), ^25^[*Clostridium*] *cellulosi* (4.5)

Footnote indicates the accession numbers for the Blast results.

^1^HQ992945.1; ^2^AJ539105.1; ^3^MH423606.1; ^4^CP001337.1; ^5^KF225561.1; ^6^MK533565.1; ^7^KY674911.1; ^8^KF999656.1; ^9^MF662230.1; ^10^MK014199.1; ^11^MG996517.1; ^12^MH989594.1; ^13^HF970590.1; ^14^LT628040.1; ^15^CP021235.1; ^16^MK541729.1; ^17^CP003200.1; ^18^MG428863.1; ^19^EU315115.2; ^20^KU163270.1; ^21^KX961722.1; ^22^MF897741.1; ^23^CP002383.1; ^24^NR_074738.1; ^25^MK431721.1.

### Changes in microbial profiles

**Figure 4:**
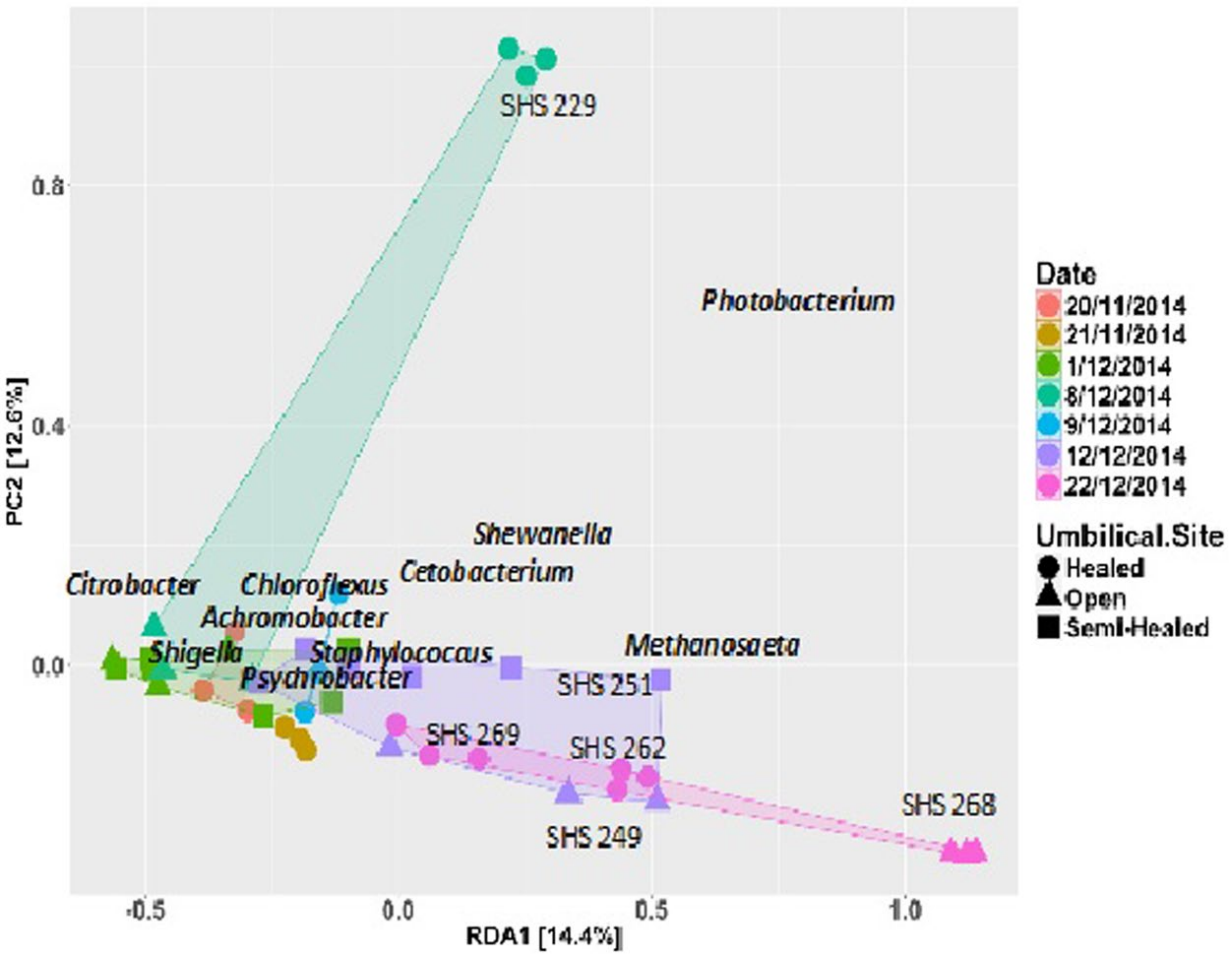
Principal Component analysis of the OTUs for individual juvenile SHS, captured on different dates (differentiated by colour) during November and December 2014, with varying degree of healing of the umbilical site (differentiated by shape), showing a shift along PC1 with samples collected after the 9^th^ of December namely SHS 249 (purple triangle), SHS 251 (purple squares, SHS 265 (pink circles close to the cluster), SHS 268 (pink triangles) and SHS 262 (pink circles furthest to the cluster). Also shown is a shift along PC2 for sample SHS 229 (mint green circles).

**Figure 5:**
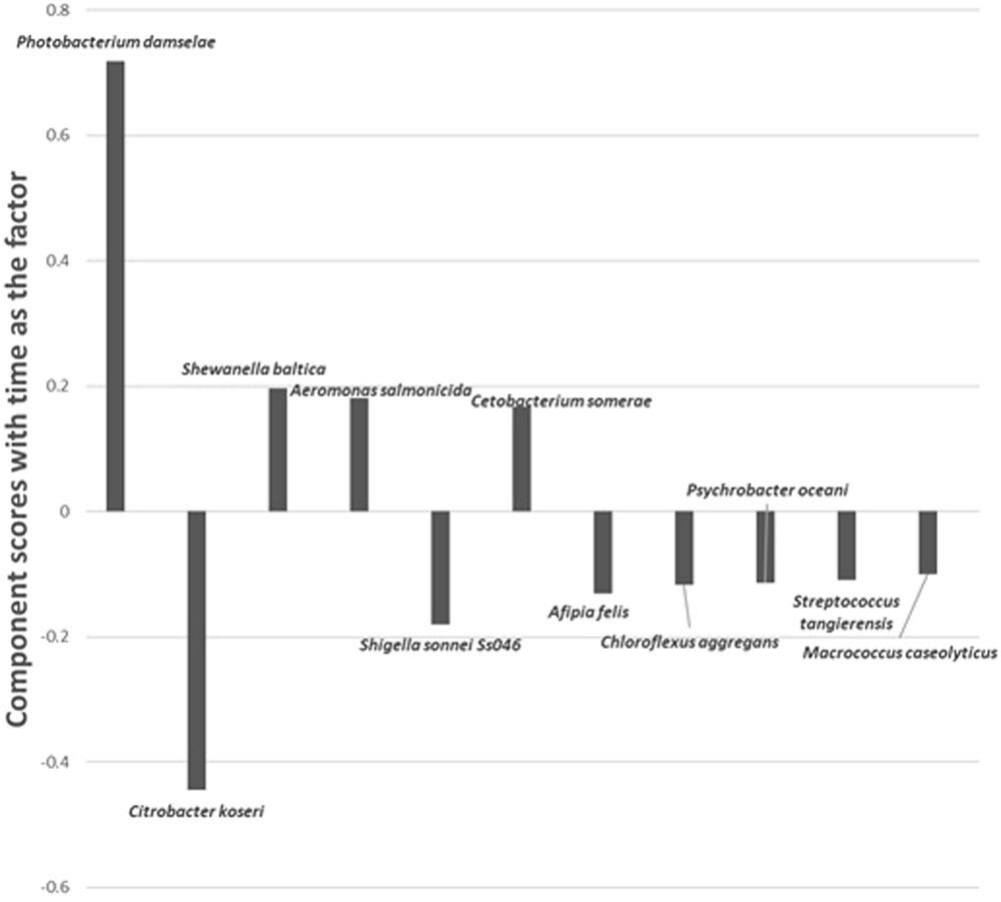
Component scores generated by Principal Component Analysis (PCA), with time of catch as the main factor, to determine the correlation between 11 major bacterial species closely related to the identified OTUs in the intestine of SHS, over a 32 days period.

The emergence of a member of the *Methanosaetaceae* family was the only archaea detected in low relative abundance in one shark captured on the 12^th^ of December 2014 (SHS 251 - 0.4%), and in 2 out of the 3 sharks captured on the 22^nd^ of December 2014 (SHS 265 - 0.5% and SHS 269 - 0.14%). These data were included in the Principal Component Analysis (PCA) against external factors of time of capture, diet, shark individuality and umbilical site (Fig. 4). Samples collected from 20^th^ of November 2014 to 9^th^ of December 2014, mostly clustered together in the PCA, while a shift to the right was observed from the 12^th^ of December 2014. Individuals SHS 249, SHS 251, SHS 262, SHS 268 and SHS 269, concurred with the dominance of *Photobacterium* sp. and, in some samples, the emergence of the archaeal methanogen. BLAST analysis related the *Methanosaetaceae* with a species of the genus *Methanosaeta* (Accession No: HM972512.2). In addition, a shift along PC2 was observed for SHS 229 sampled on the 8^th^ of December 2014, suggesting it to be radically different from all other samples. ANCOVA (Table 3) confirmed that no significant shift could be attributed to the age of the individual, estimated through the degree of healing of the umbilical scars (see Methods) and the diet at the time of capture. However, a trend was observed within the shark population (R^2^ = 0.9, p = 0.001, Table 3) and with the date it was caught (R^2^ = 0.5, p = 0.001, Table 3) (Fig. 5).

**Table 3:** ANCOVA table generated using the function “Anova” of package ‘car’ in R, to assess the significance of the impact of date of capture, shark individuality and diet, on the microbial community.

	Sum	Sq	DF	F-value	Pr (>F)
Time (date of bycatch)	*0*.*25711*	*4*	*8.4598*	*0.0001468*	***
Shark community	*1.74170*	*9*	*25.4701*	*5.916e-11*	***
Diet	*0.05412*	*1*	*7.1226*	*0.0127199*	*
Residuals	*0.20515*	*27*			

Significant Codes: 0 ‘***’ 0.001 ‘**’ 0.01 ‘*’ 0.05 ‘.’ *0*.*1* ‘ ’ *1*.

The component scores further indicated a positive correlation with time for *Photobacterium damselae* (Accession No: MH423606.1), *Shewanella baltica* (Accession No: CP002383.1), *Aeromonas salmonicida* (Accession No: LT628040.1), and *Cetobacterium somerae* (Accession No: MG428863.1). The opposite was observed for *C*. *koseri*, *Shigella sonnei*, *Afipia felis* (Accession No: HF970590.1), *Chloroflexus aggregans* (Accession No: CP001337.1), *Psychrobacter oceani* (Accession No: MH989594.1), *S*. *tangierensis*, and *Micrococcus caseolyticus* (Accession No: MG996517.1) **(**Fig. 5**)**.

## Discussion

Our investigation on the intestinal microbiome of juvenile SHS from the Rewa Delta showed a diverse microbial community, with bacteria including members of the families *Enterobacteraceae, Vibrionaceae, Propionibacteriaceae, Aeromonadaceae, Moraxellaceae, Bradyrhizobiaceae, Rhodobacteraceae, Staphylococcaceae, Streptococcaceae, Methanosaetaceae, Bradyrhizobiaceae, Fusobacteriaceae, Chlorothrixaceae, Moraxellaceae* and *Pseudomonadaceae*. These are commonly known intestinal inhabitants of terrestrial and marine vertebrate species including humans. Major members of the groups identified in this study have also previously been found in Sharpnose, Spinner and Sand sharks, although their role as gut microbiota has yet to be confirmed^23,24^. While many have been associated with digestive physiology in other species, research in such areas for shark species is limited^25^, thus making accurate determination of their function difficult.

Two OTUs accounted for a major proportion of microbial communities in all tested sharks, which BLAST analysis related to *C. koseri* and *P. damselae*, belonging to the *Enterobacteriaceae* and *Vibrionaceae* family, respectively. Both taxa have previously been associated with elasmobranch species such as bull, tiger, sharpnose, spinner and sandbar sharks^20–23^. *C. koseri* is a gram-negative anaerobic bacterium that has yet to be associated with the gut microbiome of sharks, but has been linked with their oral microbial communities and infections in humans that resulted from shark bites (as have *S. warneri*) and low water quality in the marine environment^20,26^. While *C. koseri* has been shown to have the ability to digest glucose, the importance of this function in the gut of SHS is yet to be established^27^. In comparison, *P. damselae* is believed to be a normal member of some sharks’ gut microorganisms, such as the sharpnose, spinner, and sandbar sharks^23^. In this study, both are presented as part of the intestinal habitat of the juvenile SHS due to their presence in all sharks tested. The fluctuation of dominance between these two species, over time, may reflect an environmental shift, such as changes in temperature, or food and water quality. Both species are also known opportunistic pathogens, with *P. damselae* responsible for ulcers and haemorrhagic septicaemia in brown sharks, dolphins, and shrimps^22^. It is important to note that the species level identification in this study relies on BLAST matches to partial 16S rRNA sequences; therefore, it only serves as a guidance for further investigations in an attempt to validate the presence of these potential pathogens with appropriate marker genes.

Other OTUs identified and previously seen in marine taxa were *A. felis*, *C. aggregans*, *P. adeliensis* (Accession No: AJ539105.1), *P. actiniarum* (Accession No: CP021235.1) and *S. sonnei*. While not directly linked to sharks, *A. felis* bacteria have been associated with infection in free-living amoeba and are often isolated from hospital water^28–30^. *C. aggregans* are phototrophic bacteria native to marine environment and are often associated with ‘microbial mats’^31,32^. The genus *Pontibacter* belongs to the phylum Bacteroidetes, members of which are common colonisers of fish intestine and include the isolate *P. actiniarum*, first characterised from the Sea of Japan as a gram-negative, aerobic bacterium^33^. If not present since birth, published information would suggest that these bacteria originate from preys ingested and could thus colonised the gut of the juvenile SHS sharks.

Two bacterial species dominated the intestinal microbiota of two individual shark sampled. A closer analysis with BLAST related them to *A. salmonisidas* in SHS 229, and *K. pneumoniae* in SHS 248. Both microbes are described as opportunistic pathogens associated with nosocomial respiratory tract and urinary tract infections in humans and they were observed in lesions found in the gills and intestine of a dead black tip reef shark^15,34–36^. Being two isolated cases, the probability of these bacteria being indigenous to the intestinal microbial community of juvenile SHS is quite low. A possible explanation could be that these sharks were diseased, with the said pathogens eventually dominating the gut microbial biota.

A temporal shift in community was observed in the PCA plot, towards *Methanosaeta* sp. and *P. damsalea*. Date of capture was identified as a significant factor affecting PC1. The emergence of *Methanosaeta* sp. was a major contributor to PC1. There is no indication of the presence of this methanogen as a native archaeon in the gut microbiome of any shark species studied so far. However, the role of these archaea, namely *Methanosaeta concilii*, in waste degradation, is well documented^37,38^ and its presence has previously indicated sewage or effluent contamination of waterways^7,11^. It was later revealed that, midway through this study (6^th^ of December 2014), a major sewage spill occurred in the Cunningham River, Suva, which discharged about 200 Ls^−1^ of untreated waste water into Laucala Bay. This discharge continued unabated for 18 days, until temporary control measures were implemented that led to a Government Environmental Emergency Declaration that prohibited swimming and fishing in the affected waters. Even though this discharge released untreated sewage to Laucala Bay and not to the Rewa Delta, which is about 6 km away and in the opposite direction of the trade winds and prevalent currents, the fact that we find a shift in the microbial community after the spill could be explained by two mechanisms. First, contaminated prey may have moved from Laucala Bay to the Rewa Delta via the Vunidawa River that connects Laucala Bay or second, newly born and juvenile individuals move in search of prey and fed in areas reached by the sewage^12^.

With this incident in mind, a preliminary screening of the results was carried out for other potential evidence of sewage pollution. The presence of indicator species such as *P. adeliensis* and *S. sonnei* was considered. Originally isolated from fast ice around the Antarctica region, strains of *Psychrobacter* spp. have been proven effective indicators of pollution in sites with industrial, agricultural and urban effluents^39^. It has also been isolated from marine taxa and other aquatic environments contaminated with hydrocarbons^40,41^. *S. sonnei* requires specific pH and temperature ranges to survive, and its ideal host is the human gastrointestinal tract. Shigellosis has always been associated with contaminated water, as well as contaminated seafood, and is spread easily in crowded and unhygienic conditions^42–44^. The presence of these species in all of the samples might imply that they may be indigenous to this shark species, or could also be an indicator of constant contamination of the waters of the Rewa Delta with existing pollutants associated with nearby agricultural and sewage effluents. Constant contamination from untreated sewage has been reported previously in Laucala Bay and Rewa River and is associated with two main factors. First, the Kinoya sewage treatment plant was built for a population of 77,000 persons but nowadays it supports a population of about 120,000. Second, more than 40% of the main Suva population still uses septic tanks without the ability to remove nutrients and pathogens^12^. Furthermore, some microbial growth is known to be favoured by sewage effluents that increase organic content and nutrient concentrations, as well as decreases in salinity^16^, which might explain the shift in microbe community in sharks after the sewage spill observed in juvenile SHS caught after the 9^th^ of December 2014.

## Conclusions

We have used culture-independent molecular techniques for the characterisation of the intestinal microbial community of fourteen juvenile SHS from a nursery site of the Rewa estuary in the Fiji Island of Viti Levu. This initial study provides baseline information previously lacking for this species in Fiji and in the South Pacific region. While most of the bacteria characterised have previously been identified in other shark or marine species, the bacterial community also included many known opportunistic pathogens. Determining whether these bacterial pathogens are part of the indigenous intestinal microbiome of SHS warrants further investigation. The unfortunate sewage spill that occurred during the sampling period could account for the presence of some known indicator microorganisms, namely *Methanosaeta* spp., *Shigella* spp. and *Psychrobacter* spp. It indicates that this technique can successfully identify bioindicator microorganisms associated with polluted environments.

## Methods

### Study site

The Rewa Delta (RD) (178.55°E, −18.15°S, Fig. 1) is the largest fluvial system in the Fiji Islands; it is found in the largest island of the country, Viti Levu, and it originates from the Rewa River, Fiji’s longest river. The RD is characterised by strong currents and high wave actions because of the collision between river runoff and incoming waves/tides via the reef channel. This interaction gives the RD estuarine habitat conditions, such as large fluctuating salinities, a freshwater layer, high turbidity, and tidal waves^7,45^, which collectively make 45% of the RD inaccessible for sampling.

### Sampling

Sampling sites for the current study were located on the RD, and encompassed one third of total Rewa Delta (Fig. 1). Local licensed fishermen caught the sharks studied in this experiment accidentally as bycatch during their regular fishing trips, thus no animals were intentionally sacrificed for the purpose of the current study and no IACUC or equivalent was needed. Three sharks were collected within a day of each other on three different periods, as illustrated in Table 4, except for November 2014, when only 2 sharks were available. The sharks were preserved on ice during transport for a maximum of 3 hours before immediate deep freezing at −80 °C upon return to the laboratory. Prior to DNA extraction, the intestine of each shark, including the proximal, spiral and distal region, excluding the stomach, were isolated from the specimen and any food content carefully removed and visually inspected and recorded for another study. The intestine was further cut into pieces, mixed, and separated to make 3 technical repeats for DNA extraction.

**Table 4:** Summary of the sampling records for each juvenile Scallop Hammerhead Sharks (SHS), including the stage of the umbilical scar as well as the content of the stomach and intestine, on the day of capture, removed prior to DNA extraction.

Hammerhead shark reference (SHS)	Date captured/(Collection Day)	Umbilical scar	Stomach and intestine content
169	20 Nov 2014/0	Healed	Prawn
171	21 Nov 2014/1	Healed	Eel and Prawn
190	1 Dec 2014/11	Semi-healed	Empty
194	1 Dec 2014/11	Open	Liquid
195	1 Dec 2014/11	Semi-healed	Empty
229	8 Dec 2014/18	Healed	Fish, Eel, scales and shells
230	8 Dec 2014/18	Open	Empty
239	9 Dec 2014/19	Healed	Empty
248	12 Dec 2014/22	Semi-healed	Prawn
249	12 Dec 2014/22	Open	Prawn
251	12 Dec 2014/22	Semi-healed	Prawn
265	22 Dec 2014/32	Healed	Fish
268	22 Dec 2014/32	Open	Prawn
269	22 Dec 2014/32	Healed	Fish Head

### DNA extraction

DNA extraction was performed according to the Council for Scientific and Industrial Research (CSIR) protocol for the lysis of *Corynebacterium* species, with modifications^46–48^. In brief, approximately 2 g of the mixed intestinal sample was added to 500 μl of lysis buffer (20 mM Tris-HCL at pH 8.5, 2 mM EDTA at pH 8.0), with an additional 20 mg·ml^−1^ lysozyme (Thermofischer Scientific). The mixture was then incubated for at least an hour at 37 °C in a waterbath before 50 μg ml^−1^ of Proteinase K (Thermofischer Scientific) was added to the mixture and incubated for 30 mins. Sodium dodecyl sulphate (SDS) was added (10 ml; 20% v/v) and followed by further incubation at 65 °C for 90 mins. The supernatant was collected after centrifugation at 6 000 × g for 10 mins at room temperature, mixed with an equal volume of chloroform isoamyl alcohol (24:1 v/v), and incubated for 1 min at room temperature. The mixture was centrifuged again and the resulting supernatant was precipitated with 60% of its volume of isopropanol for 60 mins at room temperature. After centrifugation at 16 000 × g for 10 mins, the resulting pellet of crude nucleic acid was washed with 500 µl of 70% cold ethanol. The extracted genomic DNA was resuspended in Tris-EDTA buffer (10 mM Tris and 1 mM EDTA, pH 7.6) and stored at −20 °C. DNA extraction was carried out in triplicates for each individual shark sample. Extracted DNA was quantified by the Qubit® 3.0 Fluorometer.

### Amplicon sequencing and data analysis

Amplification of the 16S rRNA gene region was verified using a universal primer set of 27 F (5′-AGAGTTTGATCMTGGCTCAG-3′) and 1392R (5′-ACGGGCGGTGTGTRC-3′)^37^ before submission of DNA to the Australian Centre for Ecogenomics (ACE, University of Queensland) for paired-end 16S rRNA amplicon sequencing by Illumina Miseq (Illumina Inc., USA). The amplification encompassed the V5 to V8 region of the 16S rRNA gene, using specific primers 803F (5′-TTAGANACCCNNGTAGTC-3′) and 1392Wr (5′-ACGGGCGGTGWGTRC-3′) containing Illumina adapter sequence as modified by ACE (University of Queensland, Australia).

Paired end sequencing data (ACE, University of Queensland), grouped to operational taxonomic units (OTUs) at 97% similarity, and aligned with the 16S rRNA identified sequences in the Greengenes database via the Quantitative Insights Into Microbial Ecology (QIIME) (Version 1.8.0) software package was received (ACE, University of Queensland). The resulting data were further processed in QIIME to calculate the alpha diversity including both the Shannon and Simpson indices as well as Chao 1 estimates, which is based on abundance. OTUs were further classified at the species level by BLAST analysis at 100% similarity (http://www.ncbi.nlm.nih.gov/). It is important to note that such identifications are subject to change over time should new sequences, with closer relationships, be uploaded in the database. A raw OTUs table was imported into R (v3.2.3) (R Core Team, 2015) and rarefied by function “rarefy_even_depth” of package ‘phyloseq’ (McMurdie and Holmes, 2013). The external factors of date, diet at the time of capture, shark individuality could potentially create a shift in the intestinal microbiome. In addition, at different stages of growth, juvenile SHS, as other viviparous sharks, either have the umbilical scar open, semi-open or healed. They could be more susceptible to influence from outside microorganisms if this scar is open or semi-open. Principle Component Analyses (PCAs) were performed on hellinger-adjusted OTUs tables and the above-mentioned factors as variables, with package ‘ampvis’^49^. Bar charts were generated in Microsoft Excel to illustrate the changes in the community profiles between the samples.

### Statistical and correlation analysis

Analysis of variance in mixed categorical/continuous mode (ANCOVA) was done to test the significance of environmental and host biological parameters on microbial community using the function “Anova” of package ‘car’ in R. Specifically, PC values of overall communities were used as output, date of capture was treated as a coded continuous factor, and shark individuality, diet, umbilical scar as categorical factors and numerical variables, including weight as a continuous factor. The model was tested to identify the most parsimonious, by elimination of non-significant factors to the minimum parameter. A significance threshold of 0.05 was applied for rejection of the null hypothesis. Analysis of similarity (ANOSIM) was also conducted on microbial community profile between and within triplicate analysis of individual sharks with package ‘vegan’ in R (v3.2.3) (R Core Team, 2015).
